# Matrix Metalloproteinases -8 and -9 and Tissue Inhibitor of Metalloproteinase-1 in Burn Patients. A Prospective Observational Study

**DOI:** 10.1371/journal.pone.0125918

**Published:** 2015-05-06

**Authors:** Johanna Hästbacka, Filip Fredén, Maarit Hult, Maria Bergquist, Erika Wilkman, Jyrki Vuola, Timo Sorsa, Taina Tervahartiala, Fredrik Huss

**Affiliations:** 1 Intensive Care Medicine, Department of Anesthesiology, Intensive Care Medicine and Pain Medicine, Helsinki University Hospital, Helsinki, Finland; 2 Department of Surgical Sciences, Anesthesiology and Intensive Care, Uppsala University, Uppsala, Sweden; 3 Department of Medical Sciences, Clinical Physiology, Uppsala University, Uppsala, Sweden; 4 Department of Plastic Surgery, Helsinki University Hospital, Helsinki, Finland; 5 Department of Oral and Maxillofacial Surgery, Helsinki University Hospital, Helsinki, Finland; 6 Department of Surgical Sciences, Plastic Surgery, Uppsala University, Uppsala, Sweden; 7 Burn Center, Department of Plastic- and Maxillofacial Surgery, University Hospital of Uppsala, Uppsala, Sweden; 8 Division of Periodontology, Department of Dental Medicine, Karolinska Institute, Huddinge, Sweden; INRS, CANADA

## Abstract

**Introduction:**

Matrix metalloproteinases (MMPs) -8 and -9 are released from neutrophils in acute inflammation and may contribute to permeability changes in burn injury. In retrospective studies on sepsis, levels of MMP-8, MMP-9, and tissue inhibitor of metalloproteinase-1 (TIMP-1) differed from those of healthy controls, and TIMP-1 showed an association with outcome. Our objective was to investigate the relationship between these proteins and disease severity and outcome in burn patients.

**Methods:**

In this prospective, observational, two-center study, we collected plasma samples from admission to day 21 post-burn, and burn blister fluid samples on admission. We compared MMP-8, -9, and TIMP-1 levels between TBSA<20% (N = 19) and TBSA>20% (N = 30) injured patients and healthy controls, and between 90-day survivors and non-survivors. MMP-8, -9, and TIMP-1 levels at 24-48 hours from injury, their maximal levels, and their time-adjusted means were compared between groups. Correlations with clinical parameters and the extent of burn were analyzed. MMP-8, -9, and TIMP-1 levels in burn blister fluids were also studied.

**Results:**

Plasma MMP-8 and -9 were higher in patients than in healthy controls (*P*<0.001 and *P* = 0.016), but only MMP-8 differed between the TBSA<20% and TBSA>20% groups. MMP-8 and -9 were not associated with clinical severity or outcome measures. TIMP-1 differed significantly between patients and controls (*P*<0.001) and between TBSA<20% and TBSA>20% groups (*P*<0.002). TIMP-1 was associated with 90-day mortality and correlated with the extent of injury and clinical measures of disease severity. TIMP-1 may serve as a new biomarker in outcome prognostication of burn patients.

## Introduction

Despite improvements in surgical and intensive care treatment, burn injuries continue to be associated with substantial mortality, especially in older age groups [1]. Severe burn injury triggers a systemic inflammatory response, with a rapid increase in cytokine levels [2, 3]. Different cytokine profiles are observed in survivors and non-survivors [3]. In burns larger than 20% of the total body surface area (TBSA>20%), the inflammatory response becomes generalized, leading to increased capillary permeability, fluid shifts, increased risk of sepsis, and multiple organ dysfunction [4]. Among other mediators, matrix metalloproteinases (MMPs) may contribute to the permeability changes associated with thermal injury [5].

MMPs are a group of endopeptidases that share the ability to degrade almost all components of the extracellular matrix and basement membranes [6]. Today, at least 25 different MMPs have been identified in humans [7]. MMP-8 and -9 are released from preformed granulae in neutrophil granulocytes when inflammatory stimuli are present [8, 9], and they participate in various stages of acute inflammation such as transmigration of neutrophils from the vasculature to the site of inflammation [10], degradation of matrix components, and activation and inactivation of chemokines and various cytokines [11]. They can be measured systemically almost immediately after stimuli, including bacterial lipopolysaccharide, interleukin-8, tumor necrosis factor alpha, or granulocyte colony-stimulating factor [9]. MMPs are inhibited by tissue inhibitors of metalloproteinases (TIMPs), TIMP-1 being an important inhibitor of MMP-8 and -9 [12, 13].

In sepsis, altered levels of MMP-8, MMP-9, and TIMP-1 have been associated with adverse outcome [14–17]. However, their role in the systemic inflammation associated with burn injury is poorly understood. MMP-9 and TIMP-1 levels are elevated in burn wound fluid and plasma after burn injury [18–21], and TIMP-1 levels may be higher in non-survivors [21]. However, no prospective study has specifically examined MMP-8, MMP-9, and TIMP-1 levels in the early shock phase of burn-injured patients and their association with outcome. Accordingly, we investigated the levels of MMP-8, -9, and TIMP-1 in plasma and burn blister fluid of burn-injured patients, focusing specifically on the early shock phase. We also evaluated the association between plasma MMP and TIMP-1 levels and severity of burn injury and outcome.

## Patients and Methods

### Patients and controls

The study protocol was approved by the local Institutional Review Boards (The Operative Ethics Committee of Helsinki University Central Hospital, 320/13/03/02/2011, and The Regional Ethical Review Board in Uppsala, 2011/484). All patients admitted to the burn centers of the University Hospital in Uppsala, Sweden, between March 1^st^ 2012 and February 28^th^ 2013, and Helsinki University Hospital, Helsinki, Finland, between April 1^st^ 2012 and October 31^st^ 2013, were screened for eligibility. Inclusion criteria were burn injury with a total body surface area (TBSA) burn >20% (regardless of type of burn injury) and age over 18 years. Exclusion criteria were age under 18 years, malignancy, human immunodeficiency virus infection or other immunosuppressive states, hepatitis B or C, use of immunosuppressive medication (such as systemic corticosteroids or cytostatic drugs), and tetracycline group antibiotics or bisphosphonates (known as MMP inhibitors). Burn patients with TBSA burn <20% were recruited to serve as controls with otherwise identical inclusion and exclusion criteria. We aimed for a minimum of 30 patients with >20% TBSA and 15 controls with <20% TBSA based on similar sample sizes in descriptive studies in the literature. Patients in the TBSA>20% group were regarded as having a major burn injury and those in the TBSA<20% group as having a minor or moderate burn injury [22]. Written informed consent was obtained from the patients and controls or their next of kin. In addition, a group of six healthy hospital employees was recruited to determine normal values for serum MMP- and TIMP-1 levels. The patients received standard routine care according to accepted guidelines [23].

### Samples

After receiving informed consent, blood samples from patients with >20% TBSA burns were collected as soon as possible after admission to the burn center and at 3-hour intervals thereafter in association with routine intensive care laboratory samples during the first 24 hours post-injury. Thereafter, samples were collected on days 1, 2, 3, 7, 14, and 21 after injury. Additional samples were obtained in connection with blister fluid sampling and operations. Blood samples from patients with <20% TBSA burns were taken similarly when an arterial line was clinically indicated. Otherwise, samples were taken from venipuncture at least once daily on days 0, 1, 2, 3, 7, 14, and 21. Samples were taken only during treatment at the burn center. Ten milliliters of blood was drawn into a heparinized plasma separation tube, placed on ice, and centrifuged at 1500 g for 15 minutes. The supernatant was divided into four aliquots and stored at -70°C until analysis.

To analyze MMP and TIMP-1 levels in damaged tissue, 1- to 2-ml samples of blister fluid were collected with a fine needle and syringe when blisters were present. The processing of the blister fluid was similar to that of the plasma samples. The samples were stored in the respective laboratories at a temperature of -70°C or less until they were transported for analysis.

### Other data

Patient characteristics, such as age, gender, background diagnoses, and medications, were registered. In addition, percentage of TBSA, burn trauma mechanism, and description of the depth of the burn wound on admission were recorded. Daily variables to calculate the degree of organ dysfunction were also registered. These included platelet count (reference range 150–360 x 10^9^ /l), serum creatinine (reference range 60–100 μmol/l for males, 60–90 μg/l for females), plasma bilirubin (reference range 4–20 μmol/l), maximal vasoactive medication dose, and lowest PaO_2_/FiO_2_. Sequential Organ Failure Assessment (SOFA) score was calculated on each sampling day for the TBSA>20% patients. Other laboratory data were obtained from laboratory analyses taken when clinically indicated. These included C-reactive protein (CRP) (reference range <10 mg/l), white blood cells (WBCs; reference range 3.4–8.2 x 10^9^ /l), and microbiological cultures. Patients´ weight, daily cumulative fluid dose and daily cumulative urine output for the preceding 24 hours were recorded on the days of sampling. Intensive care interventions, such as ventilator therapy, renal replacement therapy, and surgical interventions, were recorded. Septic infection within the first 72 hours was defined using a modification of the ABA definition [24]. Hyperglycemia and inability to continue enteral feeding were excluded as sepsis criteria due to the study focus on the early phase of treatment. Inhalation injury was diagnosed by bronchoscopy. Finally, the outcome (28-day and 90-day mortality) of the patients was registered.

### MMP-8, MMP-9, and TIMP-1 analysis

The levels of MMP-8 were determined by immunofluorometric analysis (IFMA) (Medix Biochemica, Kauniainen, Finland), as previously described [25]. The levels of MMP-9 and TIMP-1 were analyzed by enzyme-linked immunosorbent assay (ELISA) using commercial kits (Biotrak ELISA System; Amersham Biosciences, GE Healthcare, Buckinghamshire, UK) and according to the manufacturer’s instructions.

### Statistical analysis

Because MMP-8 and MMP-9 are dynamic markers that are released from the neutrophil granulocytes very early; 1–3 hours after the triggering insult [9], we calculated retrospectively the timing of samples using the time of burn injury instead of admission as time point 0 to be able to detect early changes in the marker levels. To demonstrate the dynamic changes in the marker concentrations, we divided the samples into several groups: 0–3 hours, 3–6 hours, 6–9 hours, 9–12 hours, 12–15 hours, 15–18 hours, 18–21 hours, 21–24 hours, 24–48 hours, 2–3 days, 3–4 days, 4–5 days, 6–8 days, 13–15 days, and 20–22 days from injury. When more than one sample from a patient was available within a time group, the one closest to the middle of the time interval was chosen. This method precluded using analysis of variance for comparisons between groups because not all patients had samples from all time-points. Thus, we performed comparisons between groups and controls and explored the association of MMP-8, MMP-9, and TIMP-1 with clinical variables and outcome at time-point 9 (24–48 hours from injury). This time-point was selected because 1) samples were available for all patients and 2) a minority of patients was expected to have sepsis at this stage. To compare MMP-8, MMP-9, and TIMP-1 concentrations over time between groups, we calculated time-adjusted means by creating areas under the curve for the samples taken within the first 72 hours from trauma. This method avoids bias from the irregularity of sampling and has previously been used in studies on hyperlactatemia and hemodynamic variation [26, 27]. The time period 0–72 hours from trauma was chosen for the following reasons: 1) the majority of control patients was likely admitted to the ward after this time period and 2) a minority of patients was expected to have sepsis at this stage. We also calculated the highest value of MMP-8, -9, and TIMP-1 during the first 72 hours from burn injury and used these values in comparisons between groups. Normality of the distribution was evaluated using the Kolmogorov-Smirnov test. Due to the non-normal distribution of the laboratory variables, the Mann-Whitney test was used for comparisons between two groups, and the Kruskall-Wallis test between three or more groups. Categorical variables were compared by using χ^2^ or Fisher´s exact test. Due to the non-normal distribution of MMP-8, -9, and TIMP-1, Spearman´s Rho test was used to test correlations. To assess the ability of TIMP-1 to predict outcome, we performed a ROC analysis and calculated areas under the curve (AUC) with 95% confidence intervals. We used stepwise backward logistic regression analysis to assess independent associations with mortality. Statistical significance was assumed at the level of *P*<0.05, except for the correlations where *P*<0.01 was considered statistically significant due to multiple variables. The analyses were performed with SPSS 20.0 for Windows (IBM, Chicago, IL, USA). The time-weighted mean/median was calculated using NCSS 8 (Kaysville, UT, USA) software.

## Results

Fifty-one patients were included in the study. Two patients were excluded from analyses; one because the time of injury was unknown and the other because the samples could not be analyzed (technical reasons). Thus, 49 patients were included in the final analyses. Thirty patients had a >20% TBSA burn and 19 had a <20% TBSA burn. Baseline characteristics and burn injury characteristics of the groups are shown in Table 1.

**Table 1:** Baseline and burn characteristics of patients.

Group	All patients, N = 49	TBSA<20% N = 19	TBSA>20% N = 30	*P*
**Age, years (IQR)**	57 (38–66.5)	57 (37–71)	57 (38–62.3)	0.81
**Gender male (%)**	35 (71.4)	12 (63.2)	23 (76.7)	0.31
**BMI**	25.4 (23.5–29.6)	28.2 (24.0–30.5)	24.8 (23.2–29.1)	0.34
**Comorbidity, N (%)**				
Cardiovascular	20 (40.8)	11 (57.9)	9 (30.0)	0.05
Psychiatric	9 (20)	4 (21.1)	5 (16.7)	0.70
Alcohol or drug abuse	2 (4.1)	3 (15.8)	1 (3.3)	0.74
Diabetes	10 (20.4)	6 (31.6)	7 (23.3)	0.52
COPD, asthma	8 (16.3)	0 (0)	2 (6.7)	0.02*
Neurological	0 (0)	0 (0)	0 (0)	
Renal	1 (2)	2 (10.5)	1 (3.3)	0.42
Miscellaneous	6 (12.2)	4 (21.1)	4 (13.3)	0.77
Metabolic	6 (12.2)		2 (6.7)	0.13
**TBSA%, (IQR)**	29.5 (15–45)	13.5 (10–15.5)	38.9 (30–50.1)	<0.001**
**Percentage of full thickness injury (IQR)**	7 (0–30)	2 (0–9)	17 (3.75–32.75)	<0.001**
**Inhalation injury, N (%)**	16 (32.7)	5 (26.3)	11 (36.7)	0.45
**Other trauma, N (%)**	0 (0)	0 (0)	0 (0)	
**Burn mechanism, N (%)**				0.01*
Scald	2 (4.1)	2 (10.5)	0	
Flame	44 (89.8)	14 (73.7)	30 (100)	
Other	3 (6.1)	3 (15.8)	0	
**Burn depth, N (%)**				0.04*
Superficial	2 (4.1)	2 (10.5)	0 (0)	
Partial dermal	6 (12.2)	3 (15.8)	3 (10.0)	
Deep dermal	21 (42.9)	7 (36.8)	14 (46.7)	
Full thickness	33 (67.3)	10 (52.6)	23 (46.9)	
**Time to hospital, hours (IQR)**	6 (2–9.38)	3.75 (1.5–12)	6.0 (2–8.4)	0.77

Categorical variables are expressed as numbers and percentages. Continuous variables are expressed as medians and inter-quartile ranges. Comparisons are between the TBSA<20% and TBSA>20% patient groups. *P*<0.05 is considered statistically significant.

* = *P*<0.05

** = *P*<0.01

TBSA% = Total body surface area percentage; BMI = Body mass index; COPD = Chronic obstructive pulmonary disease.

The patient groups were similar in baseline characteristics, except for morechronic lung diseases in the TBSA<20% group. Median time from burn injury to admission to the burn center was similar in both groups. Four patients (8.2%) had suspected and two patients (4.1%) microbiologically verified septic infection within the first 72 hours. Eight patients (16.3%) died within 90 days of the burn: 2 of 19 (10.5%) in the TBSA<20% group and 6 of 30 (20%) in the TBSA>20% group.

The median SOFA score in the TBSA>20% group at the time-point 24–48 hours was 6 (IQR 2.5–9).

**Fig 1:**
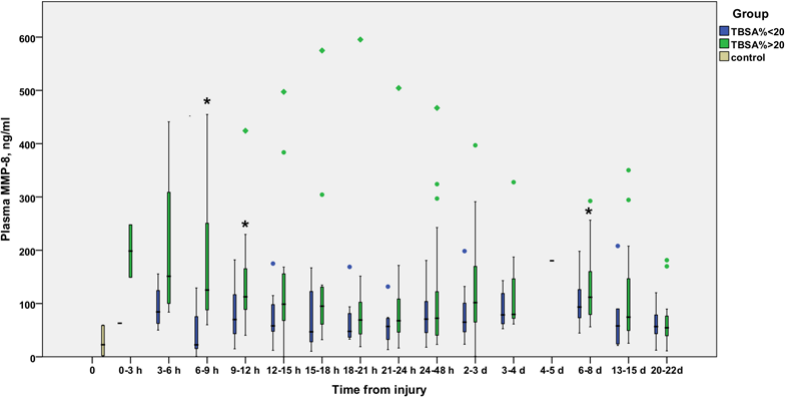
Temporal development of plasma levels of MMP-8 (ng/ml) in burn patients. Blue bars represent TBSA<20% patients and green bars TBSA>20% patients. The gray bar represents the values from six healthy controls. Samples are grouped in time intervals, calculated from the time of injury. A black asterisk indicates a statistically significant difference compared with healthy controls. A Kruskall-Wallis test was performed for each time group, except time group 12 (1 patient). After using the Bonferroni correction for multiple comparisons, significance was set at *P*<0.003. No significant differences were found between the TBSA>20% and TBSA>20% groups at any time-point.

**Fig 2:**
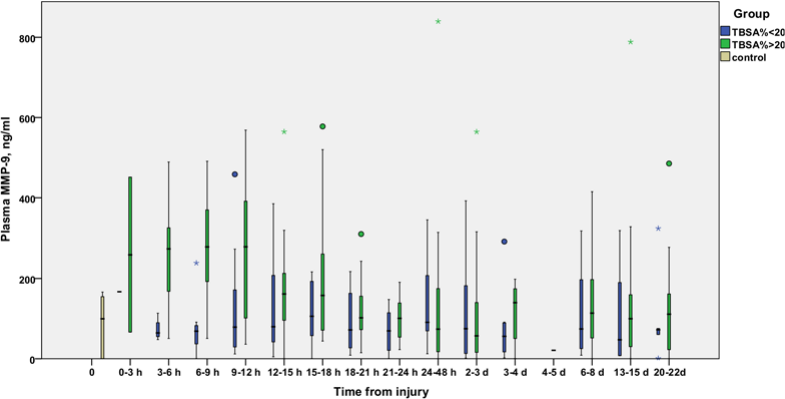
Temporal development of plasma levels of MMP-9 (ng/ml) in burn patients. Blue bars represent TBSA<20% patients and green bars TBSA>20% patients. The gray bar represents values from six healthy controls. Samples are grouped in time intervals, calculated from the time of injury. A Kruskall-Wallis test was performed for each time group, except time group 12 (1 patient). After using the Bonferroni correction for multiple comparisons, significance was set at *P*<0.003. No significant differences were found at any time-point.

**Fig 3:**
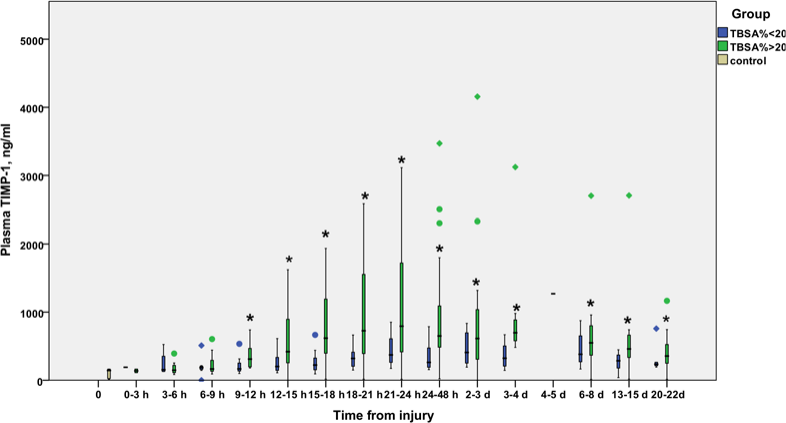
Temporal development of plasma levels of TIMP-1 (ng/ml) in burn patients. Blue bars represent TBSA<20% patients and green bars TBSA>20% patients. The gray bar represents values from six healthy controls. Samples are grouped in time intervals, calculated from the time of injury. A black asterisk indicates a statistically significant difference compared with healthy controls. A Kruskall-Wallis test was performed for each time group, except time group 12 (1 patient). After using the Bonferroni correction for multiple comparisons, significance was set at *P*<0.003.

The time courses of MMP-8 (Fig 1), MMP-9 (Fig 2), and TIMP-1 (Fig 3) are illustrated in Figs 1, 2 and 3. MMP-8 and MMP-9 levels appeared elevated in the early hours after burn injury, declining gradually to control values by 12–24 hours. TIMP-1 levels rise later, at 12 hours post-burn or later, and stay elevated throughout the study period in the TBSA>20% group.

Table 2 presents the clinical and laboratory characteristics and median values of MMP-8, -9, and TIMP-1 concentrations of the two patient groups at the time-point of 24–48 hours.

**Table 2:** Comparison of the clinical characteristics of patients in TBSA<20% and TBSA>20% groups at time interval 24–48 hours from burn injury.

	TBSA<20%, N = 19	TBSA>20%, N = 30	*P*
**SOFA**	-	6 (2.5–9)	-
**Ventilator treatment_0-72h_ N (%)**	7 (36.8)	27 (90)	<0.001**
**Dialysis_0-72h_**	1 (5.3)	2 (6.7)	0.916
**Lowest PaO_2_/FiO_2_, mmHg**	-	232.5 (183–300)	-
**Maximal noradrenaline_24h_ dose**	0 (0–0.05)	0.03 (0–0.08)	0.412
**Cumulative diuresis_24h_, ml**	1750 (1145–2200)	1585 (1064–2446)	0.75
**Cumulative fluids_24h_,ml**	2325 (1176–7093)	14764 (6800–20150)	<0.001**
**Sepsis_0-72h_**	0 (0)	6 (20)	0.115
**CRP, mg/l**	134 (32.5–176.5)	134.5 (101.7–172.5)	0.321
**WBC count, x10^9^/l**	9.6 (8.2–13.9)	8.8 (3.4–13.0)	0.314
**Creatinine, μmol/l**	71.5 (56.8–95.8)	77 (63.4–122)	0.395
**Bilirubin, μmol/l**	10 (9–16)	10 (7–18)	0.735
**Platelet count, x10^9^/l**	161 (136–217)	118 (92.5–173)	0.02*
**MMP-8, ng/ml**	70.8 (45.1–107.7)	72.4 (40.1–124.9)	0.877
**MMP-9, ng/ml**	90.9 (49.8–228.4)	73.9 (17.2–184.6)	0.32
**TIMP-1, ng/ml**	262.3 (187.9–547.9)	650.1 (472.6–1150.3)	0.001**

Categorical variables are expressed as numbers and percentages. Continuous variables are expressed as medians and inter-quartile ranges. *P*<0.05 is considered statistically significant.

* = *P*<0.05

** = *P*<0.01

SOFA = Sequential Organ Failure Assessment Score; PaO_2_/FiO_2_ = Ratio of partial oxygen pressure in arterial blood and fraction of inspired oxygen; CRP = C-reactive protein; WBC = white blood cell; MMP = matrix metalloproteinase; TIMP-1 = tissue inhibitor of metalloproteinase-1; _24h_ refers to data from the preceding 24 hours; _0-72h_ refers to data from 0–72 hours after injury.

The peak values and time-adjusted means within the time interval 0–72 hours and median times to the peak values of MMP-8, MMP-9, and TIMP-1 are shown in Table 3. As compared with healthy controls, the median peak values of MMP-8, MMP-9, and TIMP-1 were higher in the TBSA>20% group (*P*<0.001, *P* = 0.016, and *P*<0.001, respectively), whereas only median peak MMP-8 was different from healthy controls also in the TBSA<20% group (*P* = 0.019).

**Table 3:** MMP-8, MMP-9, and TIMP-1 peak and time-adjusted mean concentrations and timing of the peak levels within 72 hours of burn injury.

	TBSA<20%, N = 19	TBSA>20%, N = 30	*P*
**MMP-8_0-72h_ highest, ng/ml**	119.6 (76.4–161.7)	169.7 (104.1–331.2)	0.55
**MMP-8_0-72h_, time-adjusted, ng/ml**	66.4 (51.9–91.3)	100.9 (62.6–155.9)	0.02*
**Time to peak MMP-8, days**	0.78 (0.54–1.71)	0.74 (0.40–1.42)	0.545
**MMP-9_0-72h,_ highest, ng/ml**	211.8 (125.4–253.2)	263.6 (99.9–457.6)	0.372
**MMP-9_0-72h_, time-adjusted, ng/ml**	94.8 (52.5–145.5)	112.8 (51.2–175.4)	0.902
**Time to peak MMP-9, days**	0.64 (0.42–1.71)	0.52 (0.37–1.09)	0.492
**TIMP-1_0-72h_, highest, ng/ml**	345.2 (227.5–622.8)	815.1 (472.6–1436.2)	0.004**
**TIMP-1_0-72h_, time-adjusted, ng/ml**	280.9 (182.1–491.5)	585.5 (369.0–1252.8)	0.002**
**Time to peak TIMP-1, days**	2.09 (1.03–2.66)	1.5 (1.09–2.13)	0.119

Comparison of patients in the TBSA<20% and TBSA>20% groups. The concentrations are expressed as medians and inter-quartile ranges. TBSA% = total body surface area percentage; MMP = Matrix metalloproteinase; TIMP-1 = Tissue inhibitor of metalloproteinase-1. *P*<0.05 was considered statistically significant.

* = *P*<0.05

** = *P*<0.01

The time-adjusted means for the first 72 hours from burn injury for TBSA<20% and TBSA>20% groups differed significantly regarding MMP-8 (*P* = 0.02) and TIMP-1 (*P* = 0.002), with higher values in the TBSA>20% injured patients.

### Correlations

**Fig 4:**
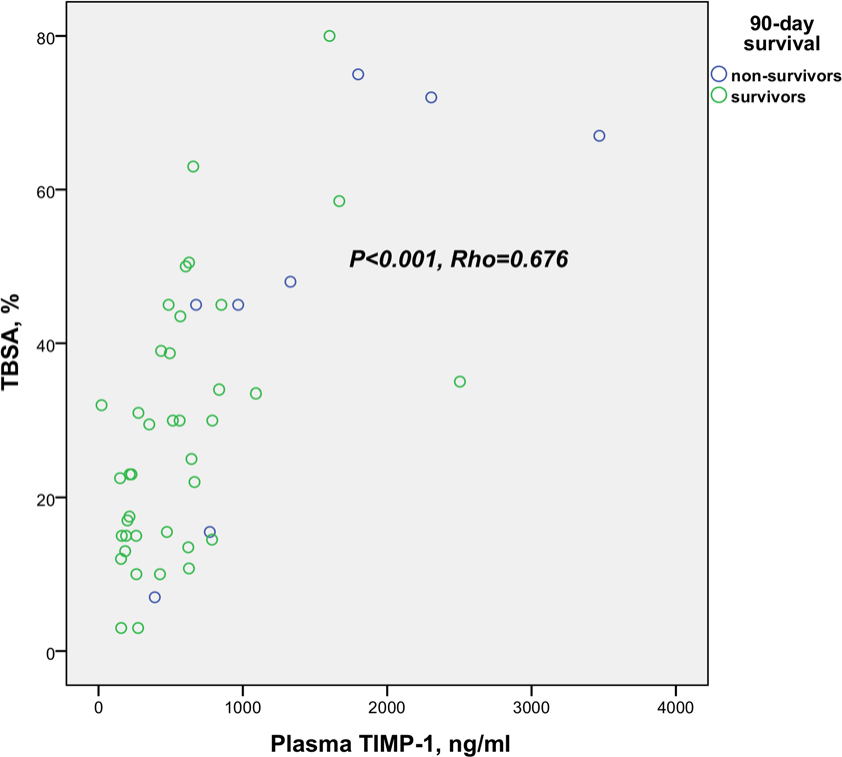
Correlation of plasma TIMP-1 and extent of burn injury. Scatter plot represents the correlation between plasma TIMP-1 (ng/ml) and extent of burn injury as TBSA percentage (all patients). Blue circles indicate 90-day non-survivors and green circles 90-day survivors.

**Fig 5:**
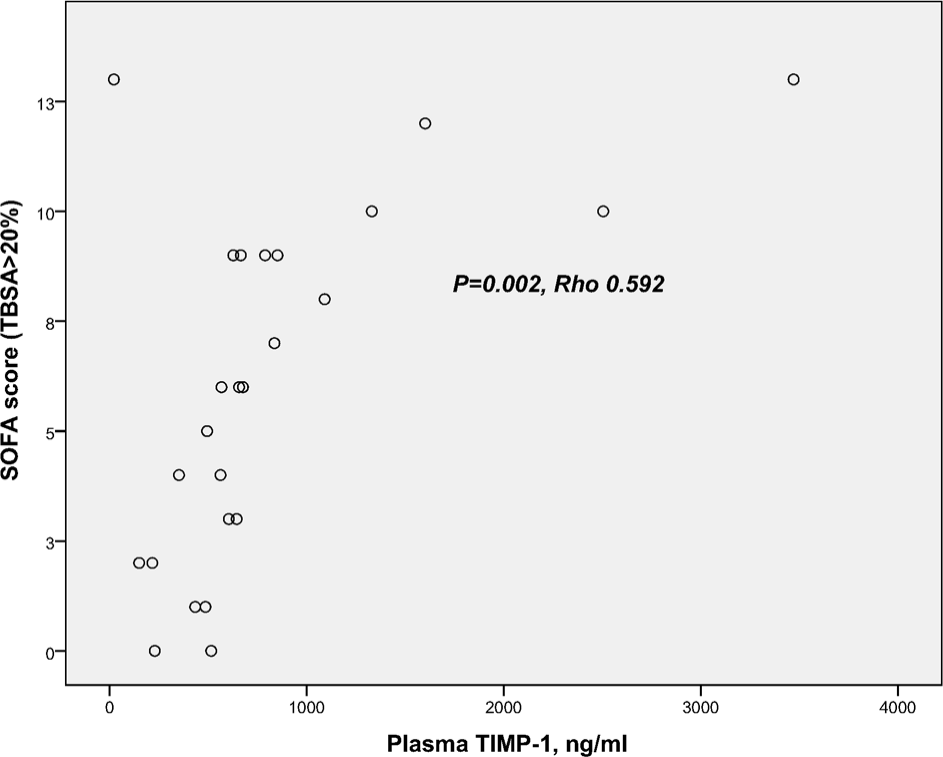
Correlation of TIMP-1 and SOFA score. Scatter plot represents the correlation between plasma TIMP-1 (ng/ml) and SOFA score in TBSA>20% patients.

Analyzed at 24–48 hours, MMP-8 and MMP-9 did not correlate with the TBSA percentage, whereas TIMP-1 showed a highly positive correlation with the extent of burn (TBSA %) (Fig 4). We also tested the correlations with the degree of intermediate injuries (TBSA percentage minus full-thickness burn percentage). This analysis revealed no correlation of MMP-8 or MMP-9 with the degree of injury. MMP-9 correlated moderately with the white blood cell count (Rho 0.503, *P* = 0.001). TIMP-1 correlated (within the TBSA>20% group) with the SOFA score (Fig 5), the highest noradrenaline dose during the preceding 24 hours (Rho 0.753, *P*<0.001), the amount of fluid administered during the preceding 24 hours (Rho 0.630, *P*<0.001), and the presence of inhalation injury (Rho 0.434, *P* = 0.002).

### Association with outcome

Median MMP-8 and MMP-9 at the time-point of 24–48 hours and the median peak values of MMP-8 and MMP-9 within 72 hours from burn injury were not different between 90-day survivors and non-survivors. Median TIMP-1 at 24–48 hours (*P* = 0.001) and median peak value of TIMP-1 (*P* = 0.004) were significantly different between 90-day survivors and non-survivors. Comparison of patient characteristics, clinical variables, and MMP-8, MMP-9, and TIMP-1 in 90-day survivors and non-survivors is shown in Table 4.

**Table 4:** Comparison of 90-day survivors and 90-day non-survivors.

	90-day survivors N = 41	90-day non-survivors N = 8	*P*
**Gender male (%)**	28 (70)	6 (85.7)	0.854
**Age, years**	56.5 (37.3–65.2)	61 (53–73)	0.038*
**TBSA%**	24 (14.6–37.8)	48 (45–72)	0.038*
**Inhalation injury, N (%)**	9 (23)	6 (75)	0.004**
**Sepsis**			0.295
Suspected	4 (9.8)		
Verified	1 (2.4)	1 (12.5)	
**MMP-8 at 24–48 h, ng/ml**	72.1 (44.9–111.5)	73.8 (33.1–168.9)	1.0
**MMP-8 highest 0–72 h, ng/ml**	136.8 (87.5–223.1)	157.4 (105.9–236.1)	0.55
**MMP-8, time-adjusted mean, ng/ml**	84.1 (53.2–126.2)	120.3 (74.2–151.8)	0.1
**MMP-9 at 24–48 h, ng/ml**	90.9 (21.3–207.4)	59.3 (26.9–229.2)	0.69
**MMP-9 highest 0–72 h, ng/ml**	243.2 (130.1–395.3)	157.7 (72.7–436.6)	0.372
**MMP-9, time-adjusted mean, ng/ml**	116.4 (52.6–154.9)	71.0 (48.2–283.3)	0.725
**TIMP-1 at 24–48 h, ng/ml**	485.6 (216.3–656.2)	1148.7 (699.7–2178.6)	0.001**
**TIMP-1 highest 0–72 h, ng/ml**	485.6 (263.0–830.2)	1237.3 (763.7–2228.4)	0.004**
**TIMP-1, time-adjusted mean, ng/ml**	388.2 (213.2–623.9)	1186.3 (606.3–1389.8)	0.002**

Demographic and clinical characteristics and MMP-8, MMP-9, and TIMP-levels are shown. Categorical variables are shown as numbers and percentages. Continuous variables are shown as medians with inter-quartile ranges. TBSA% = Total body surface area percentage; MMP = Matrix metalloproteinase; TIMP-1 = Tissue inhibitor of metalloproteinases-1. *P*<0.05 was considered statistically significant.

* = *P*<0.05

** = *P*<0.01

**Fig 6:**
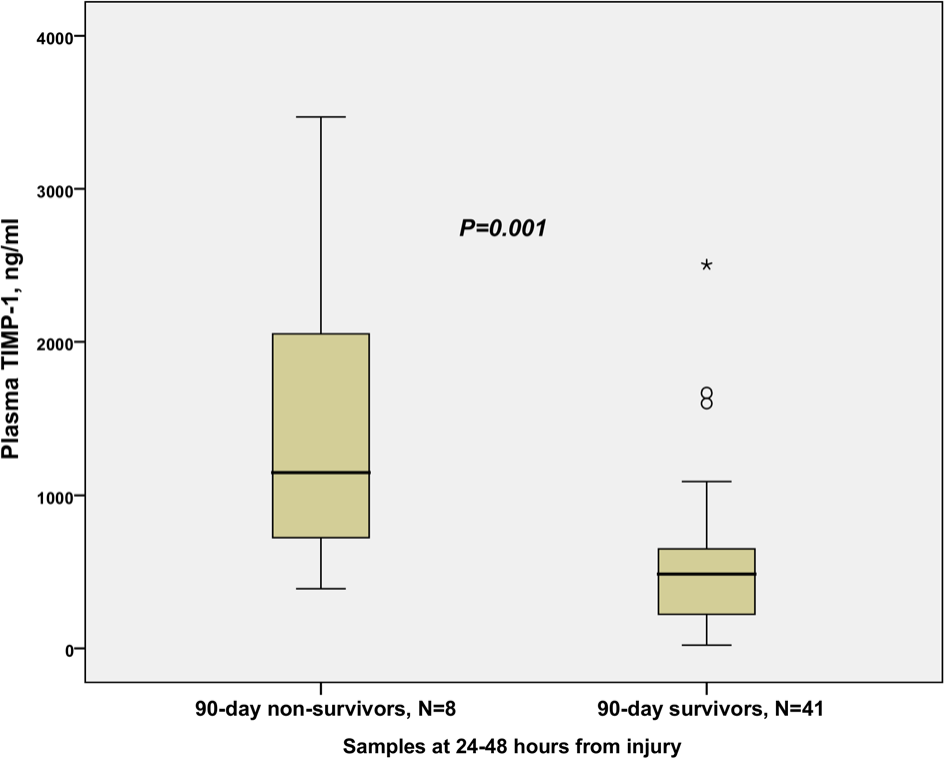
Comparison of TIMP-1 in 90-day survivors and 90-day non-survivors. Plasma levels of TIMP-1 (ng/ml) at a 24- to 48-hour time interval in 90-day survivors and non-survivors.

**Fig 7:**
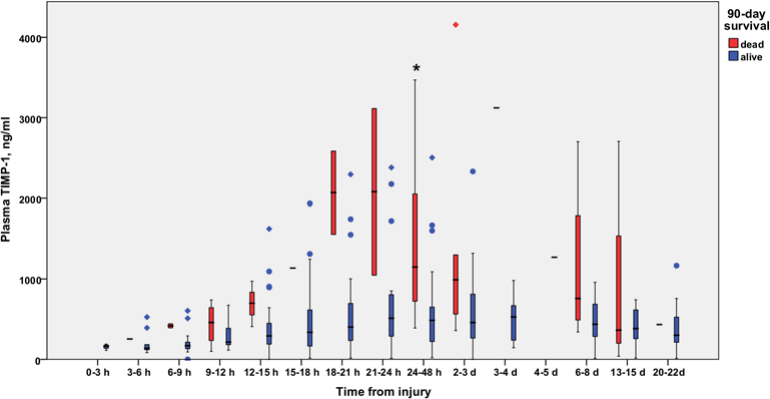
Temporal development of plasma TIMP-1 (ng/ml) in 90-day survivors and 90-day non-survivors. TIMP-1 plasma concentrations in 90-day survivors (blue) and non-survivors (red) are shown as a function of time. Samples are grouped in time intervals, calculated from the time of injury. A black asterisk indicates a statistically significant difference between groups. A Kruskall-Wallis test was performed for each time group, except time group 12 (1 patient). After using the Bonferroni correction for multiple comparisons, significance was set at *P*<0.003.

The difference in TIMP-1 concentrations at the time-point of 24–48 hours between 90-day survivors and non-survivors is illustrated in Fig 6. The time-adjusted mean TIMP-1 (*P* = 0.002*)*, but not MMP-8 (*P* = 0.109) or MMP-9 (*P* = 0.740), was significantly higher in 90-day non-survivors than in survivors. The temporal development of plasma TIMP-1 concentration in 90-day survivors and non-survivors is shown in Fig 7.

ROC analysis produced an AUC of 0.846 (95% confidence interval 0.703–0.989) (*P* = 0.002) for TIMP-1 concentration at the time-point of 24–48 hours in predicting 90-day survival. Due to the small number of non-survivors, however, we chose not to calculate any cut-off point. In stepwise logistic regression analysis, plasma TIMP-1 was independently associated with mortality (*P* = 0.03) when age, extent of burn (TBSA %), and presence of inhalation injury were in the equation.

### Blister fluid

**Fig 8:**
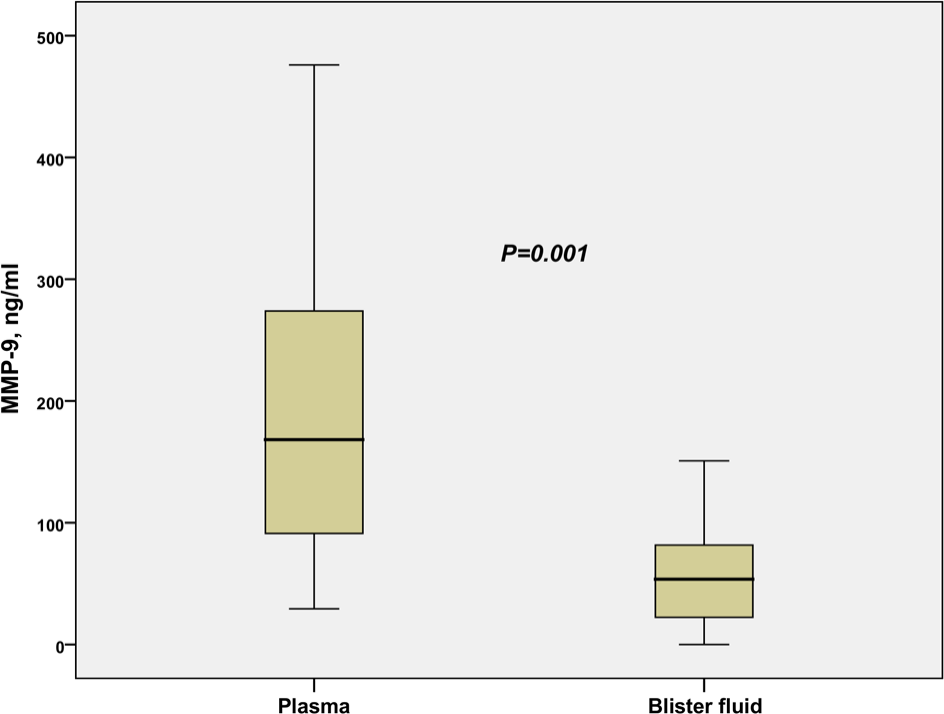
MMP-9 in plasma and burn blister fluid. The boxplot demonstrates the relationship between MMP-9 levels (ng/ml) in plasma and burn blister fluid, of patients (N = 14, five TBSA<20%, nine TBSA>20%) on admission. Patients with blister fluid samples were included in the comparison.

Blister fluid samples from 14 patients (five with TBSA<20%, and nine with TBSA>20%) were collected on admission to the burn center. Median time to sampling was 0.3 days (IQR 0.21–0.61 days). Median MMP-8, MMP-9, and TIMP-1 concentrations in the blister fluids were 24.5 ng/ml (IQR 12.0–79.5 ng/ml), 53.6 ng/ml (IQR 19.3–84.9 ng/ml), and 81.7 ng/ml (IQR 60–125.3 ng/ml), respectively. In the simultaneously collected plasma samples, the corresponding MMP-8 (*P* = 0.007), MMP-9 (*P* = 0.001), and TIMP-1 (*P* = 0.001) concentrations were significantly higher, 5.7-fold, 4-fold, and 2.9-fold, respectively. The levels of MMP-8 and MMP-9 in plasma and blister fluid did not inter-correlate. By contrast, TIMP-1 levels in plasma correlated highly with the levels in blister fluids (Rho 0.749, *P* = 0.002). Fig 8 illustrates the difference of MMP-9 concentration on admission between different body fluids.

## Discussion

In this prospective observational laboratory study, we demonstrate that plasma MMP-8 and MMP-9 are elevated in the early post-burn period in patients in the TBSA>20% group compared with healthy controls. Higher MMP-8 levels were seen in TBSA>20% patients, whereas no difference was found in MMP-9 levels between the patient groups. The highest concentrations of MMP-8 and MMP-9 are reached early, in a median of 19 and 16 hours post-injury, respectively. At 24–48 hours from injury, MMP-8 and MMP-9 levels were no longer different between the two patient groups. A similar early increase in plasma MMP-9 levels was observed recently in a retrospective laboratory analysis of burn patients [21]. In that study, MMP-9 was elevated on admission and the following day, thereafter decreasing. The patients had >15% TBSA burns, but no comparison was performed according to the severity of injury. Ulrich et al. [20] reported no elevation in serum MMP-9 until day three from injury, which may be explained by less severely injured patients in their study. They found elevated levels of MMP-9 from day three to day 21 after injury. We performed no comparisons at later stages because eventual infections could have confounded the results, as MMPs are upregulated in sepsis [14]. To the best of our knowledge, systemic MMP-8 has not been studied in burn patients before. As MMP-8 is released almost exclusively from neutrophils, the early high levels and association with the severity of injury most likely reflect the severity of systemic inflammatory reaction and activation of neutrophils.

The early increase in the MMP-8 and MMP-9 levels is likely caused by their rapid release from the pre-formed granulae in neutrophil granulocytes, which is known to occur within minutes to a few hours after inflammatory stimuli such as interleukin-8 (IL-8), tumor necrosis factor alpha (TNF-α), and granulocyte colony-stimulating factor (G-CSF) [8, 9]. TNF-α can also stimulate the synthesis of MMP-8 [25] and MMP-9 [28], the latter of which is also synthesized by cell types other than neutrophils. Also IL-1β stimulates the synthesis of MMP-8 and -9 [28, 29]. These cytokines are elevated during the first week after severe burn injury [2, 30], and high IL-8 and G-CSF are associated with worse outcome [3, 30]. MMP-8 and -9 may be downstream effectors to pro-inflammatory cytokines in the inflammatory cascade, but they also modulate the cytokine and chemokine function by cleaving IL-1β and IL-8 into more potent forms [31, 32]. After being activated, MMP- 8 and -9 cleave matrix components [6], and they have been linked with burn-associated permeability disturbance in experimental studies [5, 33]. Interestingly, this phenomenon was inhibited by a synthetic MMP inhibitor, doxycycline, in two experimental thermal injury model studies on rodents [5, 33]. However, in our study we observed no correlation of MMP levels with fluid requirement, serving as a surrogate marker for permeability disorder. We did not find an association between MMP-8 and -9 levels and organ dysfunction or outcome. In patients with sepsis, high levels of MMP-8 have been associated with increased ICU and 28-day mortality [14, 15], whereas lower levels of MMP-9 have been linked to better survival [16]. The divergence of our result may be explained by differing pathophysiology in burns and sepsis, but a more likely explanation is the small patient population and the few non-survivors in our study. Finally, the amount of active MMP-8 and -9 is probably more biologically relevant than the total enzyme levels. The presence of MMP-8 and MMP-9 does not reflect their activity [34]. With the presence of high levels of TIMP-1 in plasma, it is not unlikely that at least some of the MMPs measured may have been in an inactive form.

TIMP-1 values were elevated in the plasma of TBSA>20% patients relative to healthy controls. This is in agreement with three other studies reporting elevated TIMP-1 levels two to three days post-burn [19–21], with higher levels observed in the more severely injured patients. In our study, the median time to peak TIMP-1 concentration was 2.09 days in the TBSA<20% group and 1.5 days in the TBSA>20% group. A similar upregulation by day 2-3post-burn has been reported in other studies [19–21]. TIMP-1 levels correlated highly with the TBSA% of injury, which is in accordance with findings by Ulrich et al. [20]. TIMP-1 is expressed in various cell types, excluding neutrophils, and its synthesis is stimulated by several growth factors and cytokines such as TNF-α, IL-1β, IL-6, and IL-10 [13, 35–37]. The levels of these cytokines become elevated early post-burn [2, 3] and may thus contribute to the expression of TIMP-1 seen in our study. However, we did not measure cytokine levels.

We found for the first time in a prospective setting that plasma TIMP-1 was associated with outcome of burn patients. Plasma TIMP-1 levels at 24–48 hours, peak plasma TIMP-1 levels within 72 hours from injury, and the time-adjusted mean TIMP-1 concentration were all significantly higher in non-surviving patients, despite the small number of non-survivors in our study population. Furthermore, plasma TIMP-1 at 24–48 hours performed well in discriminating non-survivors and survivors, as measured by ROC analysis. TIMP-1 was independently associated with outcome with respect to previously known risk factors such as age, TBSA% injured, and presence of inhalation injury. TIMP-1 has been reported to be associated with mortality in severely septic patients [14, 16–17], but the mechanism is poorly understood. Of note, only one of the non-survivors in our study had suspected or verified sepsis at 24–48 hours, and thus, it is unlikely that sepsis at that time-point would explain the association with poor outcome. Recently, IL-6, an upstream regulator of TIMP-1 synthesis, was linked to outcome of burn patients [3]. It is possible that TIMP-1 is a general marker of severe inflammation or cells under distress. In the TBSA>20% group, we found a high correlation of plasma TIMP-1 levels with noradrenaline and fluid requirement, serving as surrogate measures of severity of shock and permeability disorder. Correlation with the SOFA score is likely related to the hemodynamic score, consisting mainly of the noradrenaline dose. Interestingly, catecholamines upregulate TIMP-1 *in vitro* [38].

TIMP-1 has some functions independent of its MMP inhibitory properties. It has erythroid-potentiating, cell growth-potentiating, anti-angiogenic, and pro-fibrotic capacities and is involved in steroidogenesis [13, 39]. In sepsis, it may activate neutrophils and protect them from apoptosis [40]. Association of TIMP-1 with mortality in our study does not indicate a causal relationship, nor does it provide any explanation for the mechanism, which may be associated with the MMP-inhibiting capacity or the MMP-independent properties of TIMP-1. Nevertheless, this new finding justifies a larger study assessing the value of TIMP-1 as a prognostic biomarker. Further studies are also needed to elucidate the mechanism underlying the link between TIMP-1 and unfavorable outcomes.

MMP-8, MMP-9, and TIMP-1 were detectable in burn blister fluids on admission to the burn center. Previously, MMP-9 has been found in blister and burn wound fluid in a small study, with increasing levels and activation within the first two days post-burn [18]. We are not aware of any studies reporting MMP-8 in burn blister fluids. In a study of patients with severe sepsis, suction blister fluids from severely septic patients contained similar levels of MMP-8 as burn blister fluids in our study. In that study, the blister fluid in the early stage of sepsis had higher MMP-8 and lower MMP-9 than in controls [41].

The origin of the MMPs in the blister fluid may be dual; the enzymes may diffuse from plasma and be released from invaded neutrophils or there may be local production. Plasma levels of MMPs in our study were 4- to 5-fold higher than the levels in blister fluids, and the plasma and blister fluid levels did not inter-correlate. MMPs -8 and -9 can also be produced by keratinocytes [42] and fibroblasts [25, 43], and MMPs play an important role in wound healing [44].

We found elevated levels of TIMP-1 in the blister fluids. TIMP-1 was highly positively correlated with the simultaneous levels in plasma, where the concentrations were approximately 3-fold higher. TIMP-1 has been linked to hypertrophic scar formation at later stages of burn wound healing [20]. However, we did not investigate the association with wound healing.

There are limitations in this study that must be addressed. First, the study population was relatively small, particularly with respect to non-survivors. The small patient population increases the risk of type 1 error, and thus, an association of MMP-8 and MMP-9 with outcome cannot be excluded by our findings. Nevertheless, in this population, the association of TIMP-1 with severity of injury and outcome could be detected. Second, our decision to calculate timing of samples by using injury time as the starting point can be criticized. Many studies use admission as the reference time-point, but admission times may represent a varying stage of inflammation, which eventually affects the results, particularly when investigating rapidly upregulated dynamic markers like neutrophil-derived MMPs or cytokines. In sepsis studies, this is inevitable, but in studies of trauma, the time of injury is often known. In our opinion, the calculation from injury was most certainly biologically relevant. Third, we did not measure wound MMP and TIMP levels. Rather than exploring the role of MMP-8 and MMP-9 in wound healing, our focus was on systemic inflammation in the early shock phase. Finally, we did not measure MMP-8 and -9 activities. Our negative findings of an association of MMP-8 and -9 with clinical parameters and outcome might have been different had the amount of biologically active enzyme been analyzed. Unfortunately, to the best of our knowledge, methods that specifically measure MMP-8 activity are not currently available.

## Conclusion

In summary, MMP-8 and MMP-9 were elevated early after burn injury in burn patients. They were not associated with severity of injury, clinical severity scores, or outcome in this small study. MMP-8 and MMP-9 were detectable in burn blister fluids on admission. TIMP-1 was elevated in plasma of burn patients relative to healthy controls. Higher levels were seen in plasma and blister fluid of TBSA>20% patients than in that of TBSA<20% patients. Plasma and blister fluid TIMP-1 levels were higher in 90-day non-survivors than in survivors. Plasma TIMP-1 was independently associated with 90-day mortality. TIMP-1 levels correlated with TBSA percentage of injury, fluid and noradrenaline requirement, and SOFA score. Because our patient population was small and the study was not originally designed to test TIMP-1 as a prognostic biomarker, our results are mainly hypothesis-generating. The value of TIMP-1 as a biomarker in outcome prognostication of burn patients should be investigated in a larger study. Further studies are needed to reveal the biological background for the outcome association.

## Supporting Information

S1 Dataset Patient groups, characteristics of injury, survival, timing of samples, and measured MMP-8, MMP-9, and TIMP-1 values in plasma. (SAV)

S2 Dataset Admission values of MMP-8, MMP-9, and TIMP-1 in plasma and blister fluids. (SAV)

S3 Dataset Patient and burn characteristics and clinical and laboratory variables. (SAV)
